# Association between salt intake and long-term mortality in hemodialysis patients: A retrospective cohort study

**DOI:** 10.1371/journal.pone.0260671

**Published:** 2021-12-16

**Authors:** Naoki Suzuki, Yasumasa Hitomi, Hiroya Takata, Shinji Ushiya, Masahiro Yamada, Yusuke Sakai, Takahiro Konishi, Yuuki Takeda, Yuuki Sumino, Masaya Mizo, Yoshihiro Tsuji, Masato Nishimura, Tetsuya Hashimoto, Hiroyuki Kobayashi

**Affiliations:** 1 Division of Clinical Engineering, Tojinkai Hospital, Kyoto, Japan; 2 Faculty of Health Science, Morinomiya University of Medical Sciences, Osaka, Japan; 3 Cardiovascular Division, Tojinkai Hospital, Kyoto, Japan; 4 Department of Urology, Tojinkai Hospital, Kyoto, Japan; Nagoya University, JAPAN

## Abstract

**Background:**

The association between salt intake and clinical outcomes in hemodialysis patients has been controversial. This study aimed to clarify the association between salt intake and mortality in hemodialysis patients.

**Method:**

The present study included patients who underwent hemodialysis from June 1st 2016 to May 31st 2020. Corrected salt intake by ideal body weight was the main predictor of outcomes. Ideal body weight was calculated assuming that the ideal body mass index is 22 kg/m^2^ for the Japanese population. The multivariate Cox proportional hazards model was used to determine the association between corrected salt intake and mortality, adjusting for potential confounders. The outcomes considered were all-cause mortality and cumulative incidence of cardiovascular events at year 4.

**Result:**

A total of 492 adult patients were enrolled in the study. The mean daily salt intake and corrected salt intake at baseline were 9.5 g/day and 0.17 g/kg/day, respectively. The low corrected salt intake group (< 0.13 g/kg/day) demonstrated the highest 4-year all-cause mortality. No association was observed between corrected salt intake and the cumulative incidence of cardiovascular events. In multivariate Cox proportional hazards analysis, only the group with corrected salt intake of 0.16–0.20 g/kg/day was associated with a decreased hazard risk for all-cause death compared with the low corrected salt intake group.

**Conclusion:**

The present study found that a low salt intake was associated with high all-cause mortality in hemodialysis patients. Reduced long-term survival may be attributed to malnutrition resulting from excessive salt restriction.

## Introduction

High or low sodium intake is associated with an increased risk of cardiovascular events and death in the general population [1]. For patients on hemodialysis, it is recommended that they lower their salt intake because they are at an increased risk of cardiovascular disease. European guidelines recommend a salt intake of 5–6 g/day [2], US guidelines recommend 5 g/day [3], and Japanese guidelines recommend 6 g/day [4]. Salt intake is associated with thirst and consequently with high interdialytic weight gain (IDWG) in hemodialysis patients. Some observational studies have reported that high IDWG is associated with a higher risk of all-cause and cardiovascular death and increased morbidity, such as ventricular hypertrophy and major adverse cardiac and cerebrovascular events [5–7].

However, it is also known that efforts to intensify sodium restriction can potentially increase the risk of compromising energy intake [8]. Xie et al. [9] reported that malnutrition and the risk of protein-energy wasting may occur following a sodium-restricted diet because sodium is widely used in a variety of foods. Bossola et al. [10] reported that a low daily intake of sodium is associated with an inadequately low intake of calories, proteins, minerals, trace elements, and vitamin B1. Furthermore, it was suggested that a low salt intake (< 6 g/day) was associated with an increased risk for all-cause mortality and cardiovascular death, resulting in an L-shaped association curve in Japanese patients on hemodialysis [11]. In peritoneal dialysis patients, a low dietary salt intake was an independent predictor of high all-cause and cardiovascular mortality [12]. These findings suggest that salt restriction may have a negative effect on prognosis and nutrition. In this study, we investigated the association between salt intake corrected for ideal body weight (IBW), and the incidence of cardiovascular events and all-cause mortality.

## Methods

The present study examined data from patients who underwent long-term hemodialysis in the Tojinkai group from June 1st 2016 to May 31st 2020. To be eligible for enrollment in this study, patients had to have been undergoing hemodialysis for at least 2 years at baseline. Exclusion criteria were as follows; (1) Patients on peritoneal dialysis, (2) frequency of hemodialysis was less than 3 times weekly, (3) CRP was 1.0 mg/dL or higher, and (4) patients with baseline salt intake below the 0.5 or above the 99.5 percentiles. Criterion (4) was set for the purpose of excluding outliers. (e.g., negative balance of salt intake and salt intake greater than 30 g/day).

The Ethics Committee for Human Research of Tojinkai Hospital approved this study (Number: 2021–1). The present study examined data from patients who underwent hemodialysis from June 1st 2016 to May 31st 2020. We accessed the patient’s medical records during March 2021. All data were fully anonymized before we accessed them, so that individuals could not be identified from the medical records. Written informed consent was not obtained from participants because this study was a retrospective epidemiological study. The exemption from written informed consent was approved by The Ethics Committee for Human Research of Tojinkai Hospital. The study was performed in accordance with the principles of the declaration of Helsinki, and was registered as a University Hospital Medical Information Network Clinical Trials Registry (UMIN-CTR) Clinical Trial (Unique trial Number: UMIN000043679) on 24 March 2021 (UMIN-CTR URL: http://www.umin.ac.jp/ctr/index.htm).

### Exposure/Comparison and outcomes

The main predictor of outcomes was salt intake corrected for IBW at baseline. Since salt intake correlates with dietary intake [8,9], we considered that the required salt intake varies on a patient-by-patient basis. Thus, we corrected salt intake for IBW to avoid over- or underestimating salt intake depending on body type. IBW was calculated on the assumption that the ideal body mass index (BMI) is 22 kg/m^2^ for the Japanese population [13]. In dialysis patients with anuria, sodium intake is completely balanced with water intake because the kidneys are not functioning properly. For example, 9 grams of sodium chloride intake requires subsequent intake of 1000 mL water, resulting in physiological saline [14]. Therefore, salt intake can be calculated from body weight and serum sodium concentration, pre- and post-dialysis. This method has already been validated [15,16], and was used to calculate salt intake in this study. Dialysate sodium concentration was set to 140 mEq/L. Corrected salt intake was given as follows:

$$Corrected\;salt\;intake\;[g/kg/day]=salt\;intake\;[g]/22\times Height\;[m^{2}]$$ (1)

IDWG was defined as the difference between pre-dialysis body weight at the time of the midweek dialysis session and preceding post-dialysis body weight, and the ratio of IDWG to patient’s dry weight was defined as %IDWG. The average value of corrected salt intake and %IDWG for 3 months before the start of observation was used as the baseline data. The main outcome in this study was all-cause mortality over a 4-year period. In addition, cumulative mortality over four years was analyzed using death caused by infectious disease and death caused by cardiovascular disease. Secondary outcomes were incidence of acute myocardial infarction, unstable angina, heart failure requiring hospitalization, coronary artery revascularization, stroke or cardiac sudden death. Cardiac sudden death was defined as death within 24 hours of the time at which the victim was last seen alive in a normal state of health, and cardiac diseases such as arrhythmias or acute coronary syndrome were considered the most frequent causes of death.

### Laboratory measures

Blood samples (10 ml) were collected at the time of hemodialysis every 2 weeks to measure routine biochemical and hematological factors (Hemoglobin, urea nitrogen, creatinine, calcium, inorganic phosphorus, sodium, albumin, β2-MG, and C-reactive protein). Single pool Kt/Vurea, and normalized protein catabolism rate (nPCR) were calculated using the formula of Shinzato et al. [17]. The average value for 3 months before the start of observation was used as the baseline.

### Statistical analysis

We first grouped patients by the interquartile range of corrected salt intake: Q1 (< 0.13 g/kg/day), Q2 (0.13- < 0.16 g/kg/day), Q3 (0.16- < 0.20 g/kg/day), and Q4 (> 0.20 g/kg/day). Baseline characteristics were compared using analysis of variance or the Kruskal-Wallis test for continuous variables and the chi-square test for categorical variables.

We examined all-cause survival using the Kaplan-Meier method and the log-rank test adjusted by the Bonferroni method. Prognosis was assessed using three multivariate Cox proportional hazards models. The model adjusted by nutrition-related factors (age, male sex, BMI, albumin, and nPCR) was designated “adjusted model 1”; this first model adjusted by adding cardiovascular risk factors (%IDWG, pre-dialysis systolic blood pressure, and past cardiovascular events) was designated “adjusted model 2”; and model 2 adjusted by adding other factors (presence of diabetes mellitus, dialysis duration and Kt/V) was designated “adjusted model 3”. The cumulative mortality caused by infectious disease and cardiovascular disease was calculated using the Gray test, treating death other than death due to infectious disease and cardiovascular disease as a competing risk. The cumulative incidence of cardiovascular events was also calculated using the Gray test, treating death other than cardiovascular death as a competing risk. In this study, we performed a nested, case-control study using sub-groups of the same age distribution because the age distribution groups Q1–4 are different. The nested case-control study compared corrected salt intake in the survivor and non-survivor groups. Propensity score matching was performed to select patients in each group, using age and dialysis duration as confounders. Corrected salt intake was compared using Student’s t test.

Normally distributed values were expressed as mean ± the standard deviation, while the other values are expressed as the median and interquartile range. P values < 0.05 were considered significant. All statistical analyses were performed with R version 4.0.3.

## Results

**Fig 1:**
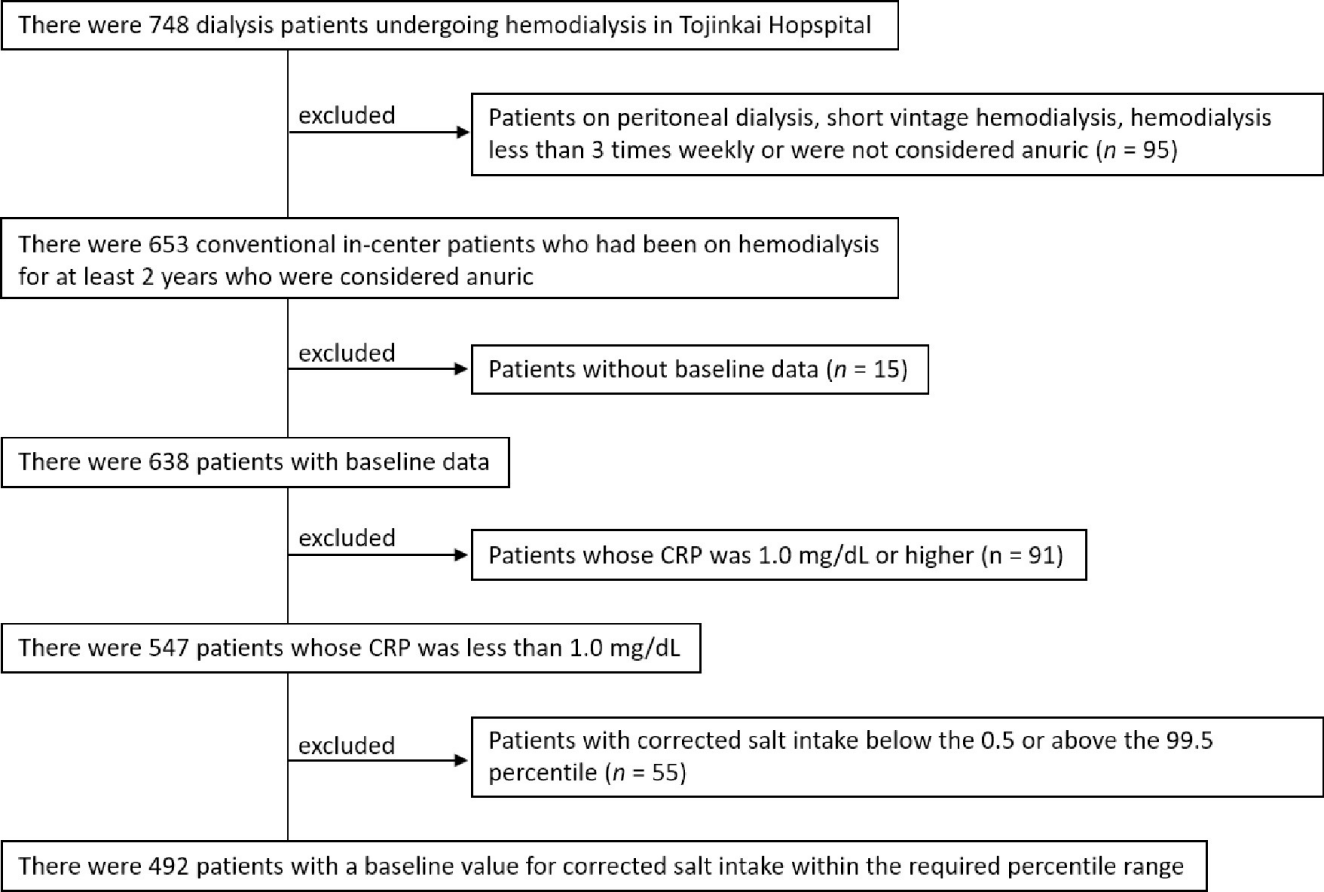
Flowchart of the study enrolment process. CRP, C-reactive protein.

A total of 492 adult patients were enrolled in the study. The process by which patients were enrolled is shown in Fig 1. Baseline characteristics are shown in Table 1. For the overall population, the mean age was 67.6 years, 57.1% were men, 40.0% had diabetes, and 38.8% had past cardiovascular events. At baseline, the mean daily salt intake and corrected salt intake were 9.5 g/day and 0.17 g/kg/day, respectively. There were 434 patients (88.2%) with a salt intake of > 6 g/day. Generally, patients with a high corrected salt intake were younger, and had a shorter dialysis duration. Patients with high corrected salt intake had higher dry weight, BMI, %IDWG, blood pressure, hemoglobin, urea nitrogen, creatinine, calcium, inorganic phosphorus, sodium, albumin, and nPCR. Of the 492 participants, 107 (21.7%) died and of these, 103 (20.9%) were attributable to cardiovascular events during the 4-year observation period. The causes of death in each group are shown in Table 2. Mortality due to infectious diseases (e.g., pneumonia, sepsis) was the highest, and tended to be higher in the group with lower corrected salt intake (shown in Table 2).

**Table 1:** Characteristics of the study participants at baseline according to corrected salt intake.

Variables	Overall (n = 492)	Corrected salt intake rate by ideal body weight, g/kg/day				p value
		Q1 (< 0.13) (n = 43)	Q2 (0.13 to < 0.16) (n = 116)	Q3 (0.16 to < 0.20) (n = 145)	Q4 (> 0.20) (n = 121)	
Age, years	67.6±11.9	71.2±12.2	68.9±11.0	67.0±11.2	63.9±12.4	< .0001
male, n (%)	281 (57.1)	64 (58.2)	62 (53.4)	86 (59.3)	69 (57.0)	0.8080
Hemodialysis vintage, years	11.0 (5.0–17.0)	11.0(6.0–18.0)	10.5(5.0–18.0)	11.0 (6.0–16.0)	9.0 (5.0–16.0)	0.5379
Diabetes mellitus, n (%)	197 (40.0)	70 (63.6)	67 (57.8)	90 (62.1)	68 (56.2)	0.6070
Past cardiovascular events, n(%)	191 (38.8)	50 (45.9)	42 (35.9)	59 (40.7)	40 (33.1)	0.2029
Dry weight, kg	56.3±12.6	50.3±8.7	53.4±10.2	57.3±10.6	63.4±15.8	< .0001
Ideal body weight, kg	56.7±6.9	56.1±6.7	56.0±6.6	57.3±6.3	57.3±7.8	0.2430
Body mass index, kg/m^2^	21.8±3.6	19.8±2.8	21.0±3.0	22.0±2.9	24.2±4.1	< .0001
Ratio of IDWG to dry weight, %	2.5±1.5	2.3±1.5	2.2±1.4	2.5±1.5	3.0±1.7	0.0005
Predialysis systolic blood pressure, mmHg	139.8±20.6	135.6±20.9	139.0±20.8	140.8±18.3	143.4±22.1	0.0301
Predialysis diastolic blood pressure, mmHg	72.3±15.0	68.5±14.8	71.0±14.0	72.8±14.9	76.3±15.5	0.0007
Salt intake, g/day	9.5±3.1	5.8±1.3	8.1±1.0	10.3±1.3	13.2±2.6	< .0001
Hemoglobin, g/dl	10.8±1.0	10.5±1.1	10.7±0.9	10.7±1.0	11.1±1.2	0.0016
Urea nitrogen, mg/dl	59.8±13.6	54.9±13.4	59.1±13.9	61.5±13.4	62.9±12.6	< .0001
Creatinine, mg/dl	10.4±2.5	9.5±2.5	10.1±2.3	10.8±2.3	11.2±2.5	< .0001
Calcium, mg/dl	8.8±0.7	8.7±0.7	8.8±0.6	8.8±0.6	8.9±0.7	0.0748
Inorganic phosphorus, mg/dl	4.7±1.0	4.5±1.0	4.7±0.9	4.8±1.0	4.9±1.1	0.0123
Sodium, mEq/L	138.2±3.1	136.4±3.0	138.6±2.4	139.5±2.4	140.9±2.6	< .0001
Albumin, g/dl	3.7±0.3	3.6±0.4	3.7±0.3	3.8±0.3	3.8±0.3	< .0001
β2-microglobulin, mg/l	28.8±6.7	30.1±7.0	29.0±7.5	28.3±6.7	28.0±5.1	0.0813
C-reactive protein, mg/dl	0.09 (0.04–0.22)	0.12 (0.05–0.31)	0.08 (0.04–0.19)	0.09 (0.04–0.19)	0.13 (0.05–0.26)	0.0636
Single pool Kt/V urea	1.85±0.36	1.84±0.44	1.85±0.31	1.85±0.31	1.85±0.33	0.9880
Normalized protein catabolism rate, g/kg/day	0.89±0.16	0.82±0.15	0.88±0.16	0.91±0.15	0.93±0.15	< .0001

**Table 2:** Causes of death in groups by corrected salt intake.

	Overall (n = 107)	Corrected salt intake rate by ideal body weight, g/kg/day				p value
		Q1 (< 0.13) (n = 43)	Q2 (0.13 to < 0.16) (n = 26)	Q3 (0.16 to < 0.20) (n = 18)	Q4 (> 0.20) (n = 20)	
infectious disease, n (%)	37 (34.6)	21 (48.8)	8 (30.8)	4 (22.2)	4 (20.0)	0.079
Cardiovascular disease, n (%)	28 (26.2)	10 (23.3)	7 (26.9)	3 (16.7)	8 (40.0)	0.410
Cancer, n (%)	15 (14.0)	5 (11.6)	5 (19.2)	4 (22.2)	1 (5.0)	0.354
Intestinal ischemia, n (%)	5 (4.7)	0 (0.0)	3 (11.5)	1 (5.6)	1 (5.0)	0.091
Unknown, n (%)	8 (7.5)	3 (7.0)	1 (3.8)	2 (11.1)	2 (10.0)	0.777
Others, n (%)	14 (13.1)	4 (9.3)	2 (7.7)	4 (22.2)	4 (20.0)	0.335

**Fig 2:**
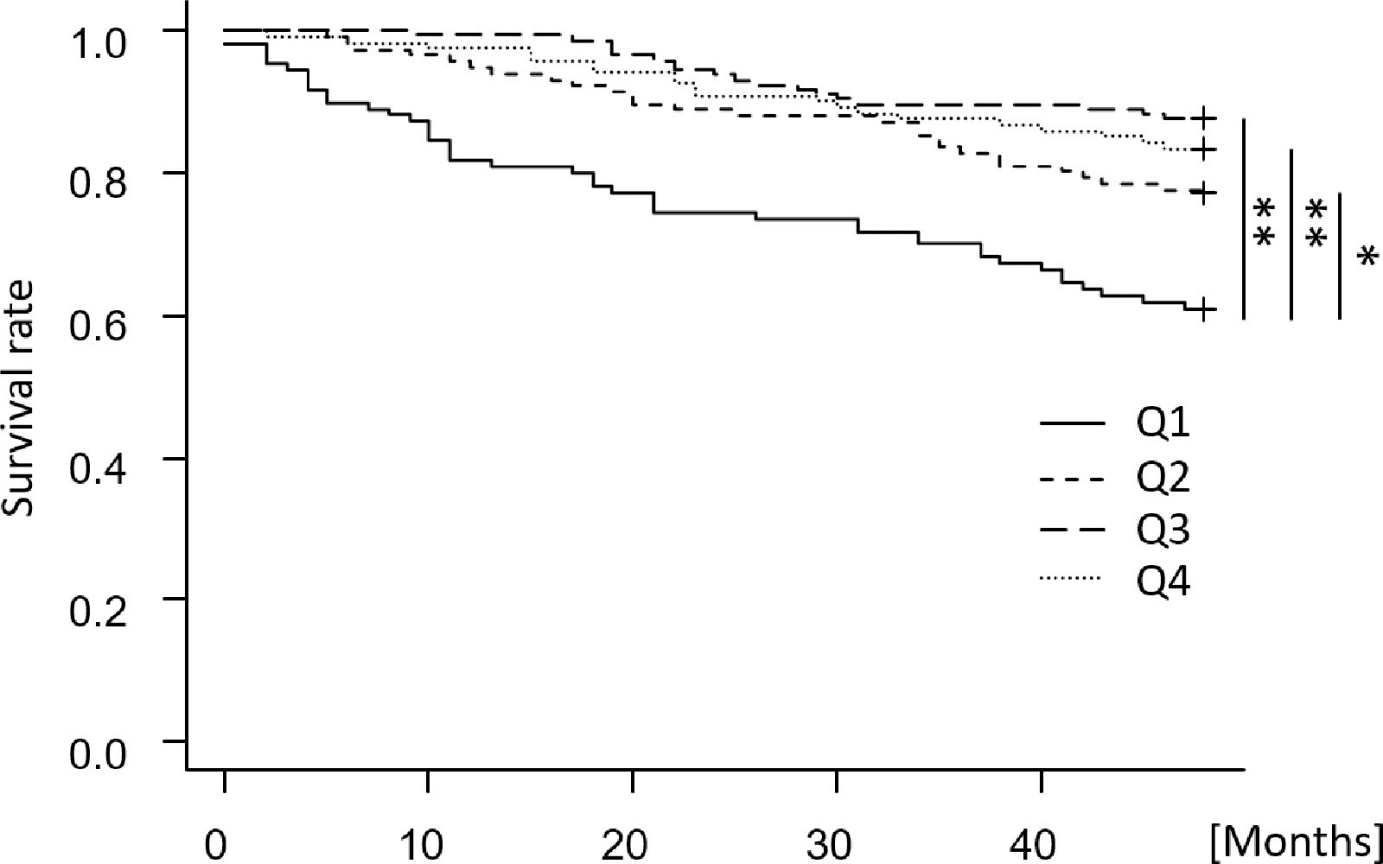
Kaplan-Meier analyses of all-cause survival according to corrected salt intake in four-year follow-up. Categories of corrected salt intake: Q1, < 0.13 g/kg/day; Q2, 0.13 to < 0.16 g/kg/day; Q3, 0.16 to < 0.20 g/kg/day; Q4, ≥ 0.20 g/kg/day. Log-rank test of differences P < 0.001. * represents P < 0.05. ** represents P < 0.01.

**Fig 3:**
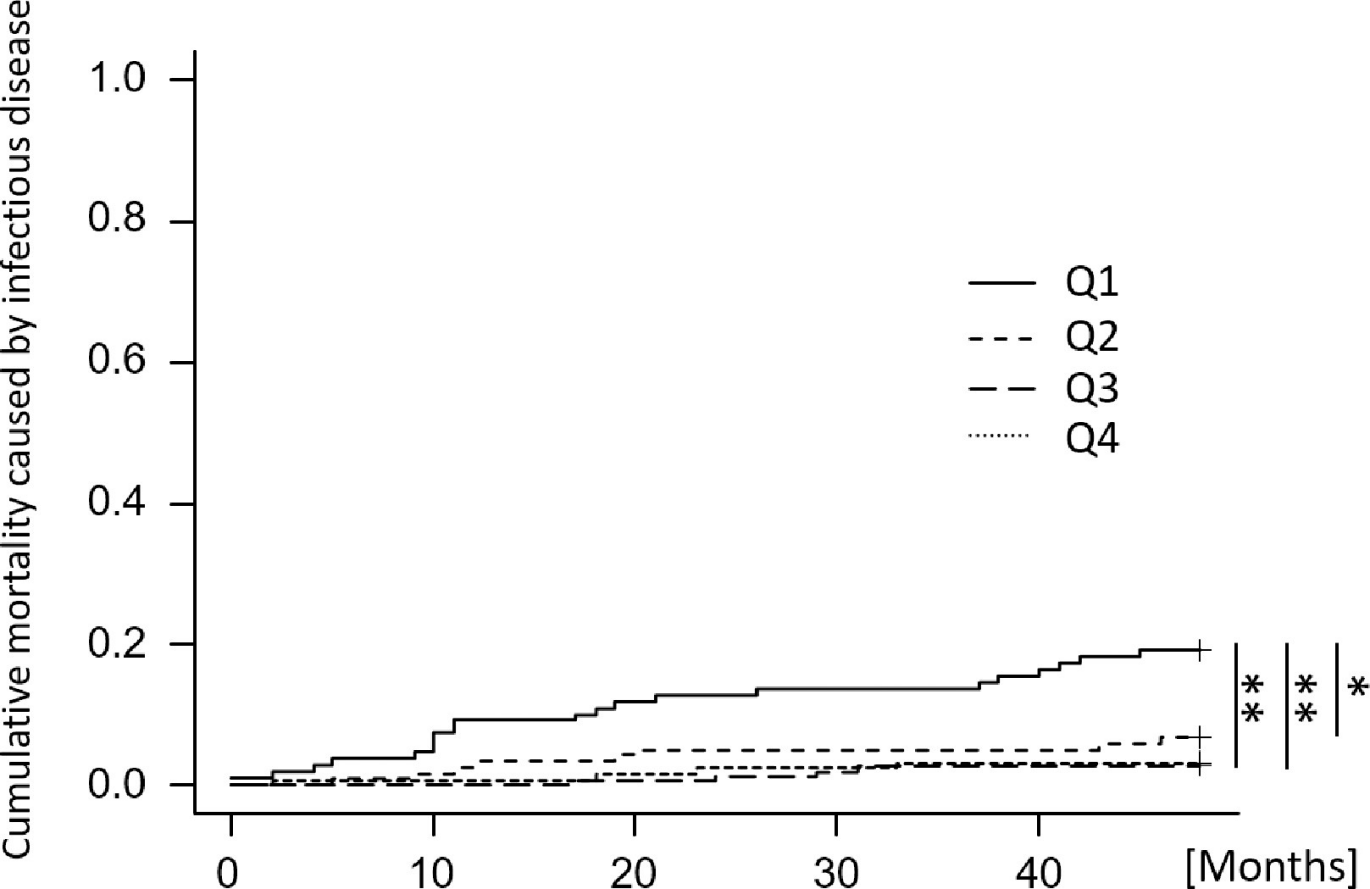
Cumulative mortality caused by infectious disease according to corrected salt intake with four-year follow-up. The cumulative mortality was calculated using the Gray test, treating deaths other than death due to infectious disease as a competing risk. Categories of corrected salt intake: Q1, < 0.13 g/kg/day; Q2, 0.13 to < 0.16 g/kg/day; Q3, 0.16 to < 0.20 g/kg/day; Q4, ≥ 0.20 g/kg/day. Gray test of differences P < 0.001. * represents P < 0.05. ** represents P < 0.01.

**Fig 4:**
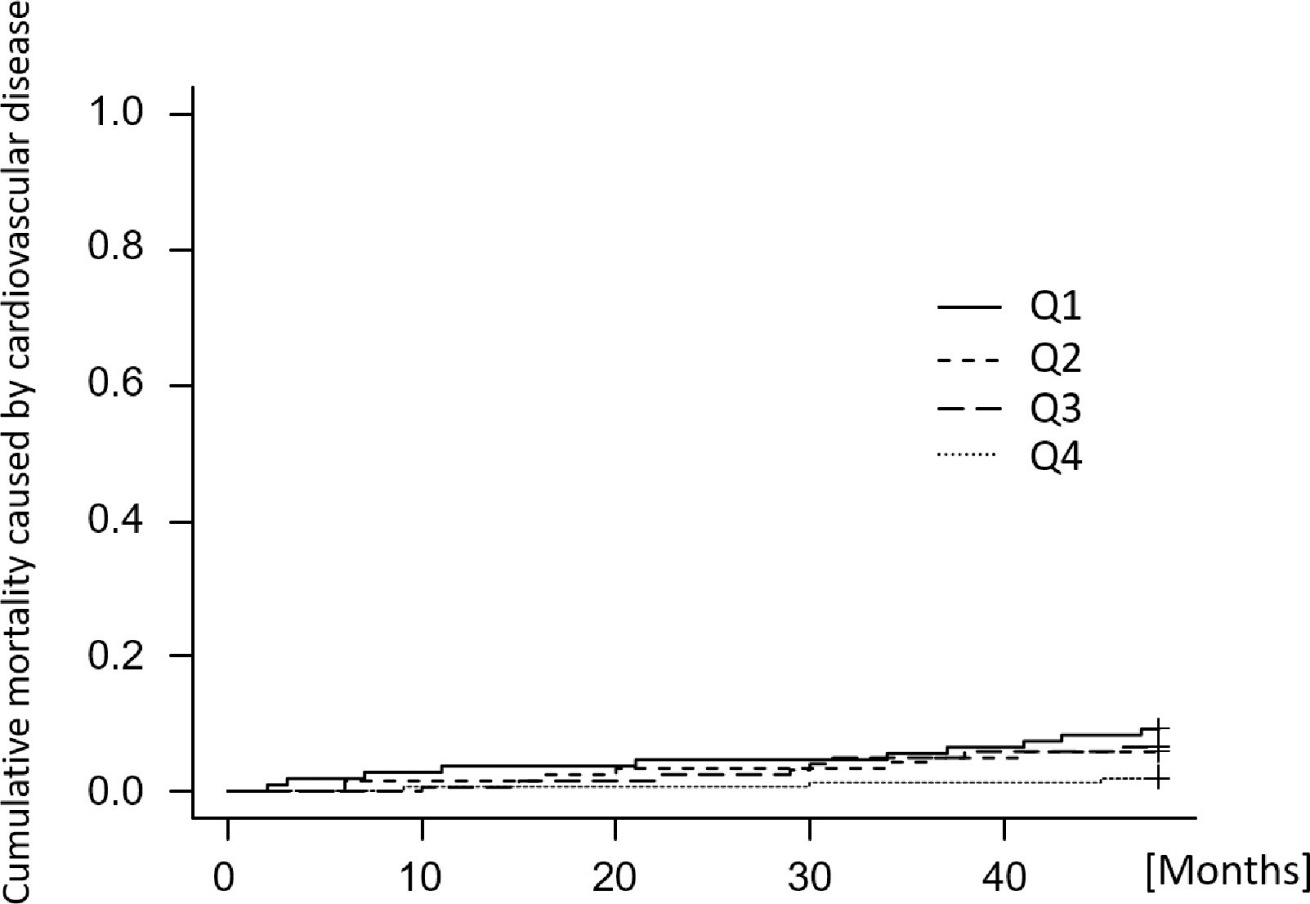
Cumulative mortality caused by cardiovascular disease according to corrected salt intake with four-year follow-up. The cumulative mortality was calculated using the Gray test, treating deaths other than death due to cardiovascular as a competing risk. Categories of corrected salt intake: Q1, < 0.13 g/kg/day; Q2, 0.13 to < 0.16 g/kg/day; Q3, 0.16 to < 0.20 g/kg/day; Q4, ≥ 0.20 g/kg/day. Gray test of differences P < 0.102.

In the Kaplan-Meier survival analysis, the 4-year all-cause survival rate was significantly higher in the Q2–4 groups than in the Q1 group (Q1: 60.9%, vs. Q2: 76.7%, vs. Q3: 87.6%, vs. Q4: 83.5%) (shown in Fig 2). In adjusted model 1 of the multivariate Cox proportional hazards models, all nutritional factors were inversely associated with all-cause death (shown in Table 3). In adjusted model 2, %IDWG and past cardiovascular events were positively associated with an increased hazard risk. In adjusted model 3, age, %IDWG, past cardiovascular events and dialysis duration were positively associated, and albumin, nPCR and single pool Kt/V were inversely associated with all-cause death. In all models, the Q3 group was associated with a decreased hazard risk for all-cause death compared to the Q1 group. Cumulative mortality caused by infectious disease and cardiovascular disease is shown in Figs 3 and 4, respectively. The cumulative mortality caused by infectious disease over four years was significantly higher in the Q1 group than in the Q2–4 groups (Q1: 19.2%, vs. Q2: 6.8%, vs. Q3: 2.7%, vs. Q4: 3.3%), while there was no significant difference in the cumulative mortality caused by cardiovascular disease (Q1: 9.2%, vs. Q2: 6.0%, vs. Q3: 2.1%, vs. Q4: 6.6%).

**Table 3:** Result of multivariate Cox proportional hazards analysis.

	Unadjusted			Adjusted model 1			Adjusted model 2			Adjusted model 3		
	HR	95% CI	p value	HR	95% CI	p value	HR	95% CI	p value	HR	95% CI	p value
Age	1.07	1.05–1.09	< .0001	1.04	1.01–1.06	< .0001	1.03	1.01–1.05	0.0026	1.04	1.01–1.06	0.0005
Male	0.89	0.60–1.31	0.5550	0.86	0.59–1.30	0.5164	0.94	0.63–1.41	0.7992	1.02	0.66–1.57	0.9276
Body mass index	0.88	0.82–0.93	< .0001	0.91	0.85–0.97	0.0081	0.94	0.87–1.01	0.0954	0.95	0.88–1.03	0.2538
Albumin	0.09	0.05–0.16	< .0001	0.22	0.11–0.41	< .0001	0.24	0.12–0.46	< .0001	0.28	0.14–0.56	0.0033
Normalized protein catabolism rate	0.05	0.01–0.21	< .0001	0.17	0.04–0.68	0.0120	0.09	0.02–0.39	0.0013	0.11	0.02–0.48	0.0024
Predialysis systolic blood pressure	0.99	0.98–1.00	0.1362	-	-	-	0.99	0.99–1.00	0.8854	0.99	0.99–1.00	0.9380
Ratio of absolute IDWG to dry weight	1.08	0.95–1.23	0.1896	-	-	-	1.19	1.01–1.40	0.0314	1.22	1.03–1.44	0.0185
Past cardiovascular events	3.06	2.47–3.79	< .0001	-	-	-	1.78	1.18–2.68	0.0058	1.58	1.03–2.40	0.0329
Diabetes mellitus	1.52	1.04–2.22	0.0292	-	-	-	-	-	-	1.28	0.80–2.02	0.2918
Hemodialysis duration	1.01	0.99–1.03	0.0868	-	-	-	-	-	-	1.03	1.01–1.06	0.0015
Single pool Kt/V urea	0.34	0.18–0.64	0.0009	-	-	-	-	-	-	0.47	0.23–0.97	0.0418
Corrected salt intake for ideal body weight												
Q1(< 0.13)	Reference			Reference			Reference			Reference		
Q2(0.13 to <0.16)	0.48	0.29–0.78	0.0033	0.80	0.42–1.19	0.1983	0.74	0.44–1.24	0.2669	0.73	0.43–1.22	0.2304
Q3(0.16 to <0.20)	0.25	0.14–0.44	< .0001	0.61	0.24–0.88	0.0204	0.48	0.25–0.93	0.0295	0.48	0.25–0.91	0.0262
Q4(≥0.20)	0.34	0.20–0.59	< .0001	1.06	0.32–1.39	0.2875	0.76	0.36–1.57	0.4611	0.66	0.31–1.38	0.2749

Outcome was all-cause mortality; predictors were categories of corrected salt intake, respectively. HR, hazard ratio; 95% CI, 95% confidence interval. Model 1 was adjusted for nutritional factors. Model 2 was adjusted for cardiovascular risk factors in addition to the adjustments of model 1. Model 3 was adjusted for other factors in addition to those used in model 2.

**Fig 5:**
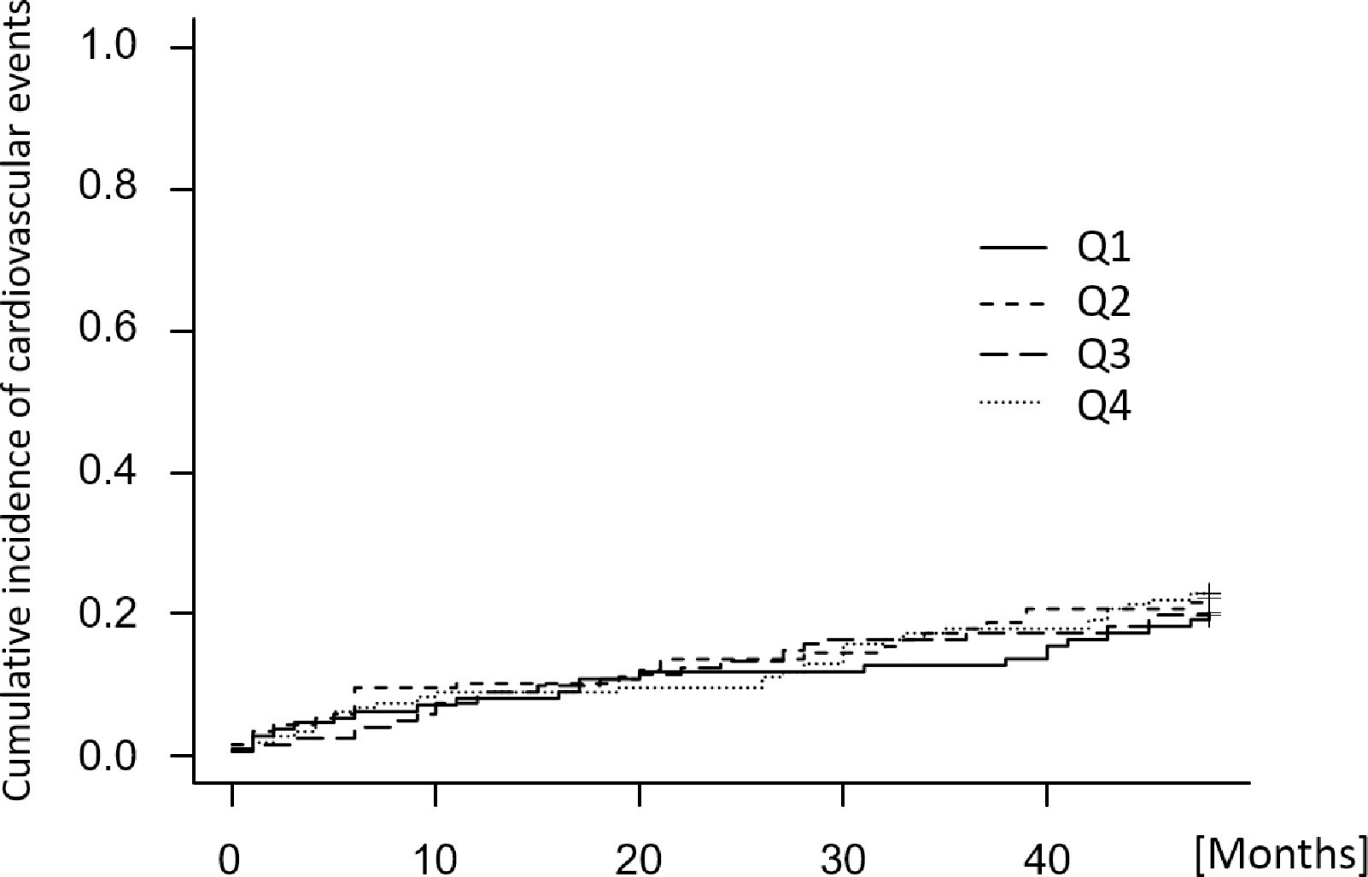
Cumulative incidence of cardiovascular events according to corrected salt intake with four-year follow-up. The cumulative incidence of cardiovascular events was calculated using the Gray test, treating death other than cardiovascular death as a competing risk. Cardiovascular events were defined as acute myocardial infarction, unstable angina, heart failure requiring hospitalization, coronary artery revascularization, stroke or cardiac sudden death. Categories of corrected salt intake: Q1, < 0.13 g/kg/day; Q2, 0.13 to < 0.16 g/kg/day; Q3, 0.16 to < 0.20 g/kg/day; Q4, ≥ 0.20 g/kg/day. Gray test of differences P = 0.993.

The cumulative incidence of cardiovascular events is shown in Fig 5. At 4-year follow-up, the cumulative incidence estimates were 20.0%, 21.6%, 21.4%, and 20.7% for the Q1, Q2, Q3, and Q4 groups, respectively. There was no significant difference in the cumulative incidence.

The nested case-control study enrolled 104 patients each in the survivor and non-survivor groups. In the survivor and non-survivor groups, the ages were 73.9 ± 9.0 and 74.0 ± 9.0 years (p = 0.963), and dialysis durations were 12.0 (6.0–20.3) and 11.0 (5.8–18.0) years (p = 0.767), respectively. The proportions of males and people with diabetes were not significantly different between the two groups. Corrected salt intake in the survivor and non-survivor groups was 0.17 ± 0.04 g/kg/day and 0.15 ± 0.05 g/kg/day (p = 0.008), respectively, and corrected salt intake in the non-survivor group was significantly lower (p = 0.008).

## Discussion/Conclusion

This study showed that low corrected salt intake (< 0.13 g/kg/day) was associated with high four-year all-cause mortality. In multivariate Cox proportional hazards analysis, a corrected salt intake of 0.13–0.20 g/kg/day was associated with higher survival at 4 years. In a study of 88,115 dialysis patients in Japan, 1-year all-cause mortality was highest in the low salt intake group (< 6 g/day), and was lowest in the group with a salt intake of 8–9.99 g/day [11]. In this study, the mean salt intake of the Q3 group was 10.3 ± 1.3 g/day which was similar to the group with the lowest mortality in the aforementioned study. We also found that a low corrected salt intake (< 0.13 g/kg/day) was associated with high four-year mortality due to infectious disease (shown in Fig 3). The principal strength of the study is that we showed a relationship between salt intake and cause of death, not identified in previous reports.

There is an association between salt intake and nutrition intake. Bossola et al. [10] showed that patients with a daily sodium intake lower than 1,500 mg, in comparison with those with an intake of 1,500 mg or more, were at a significantly greater risk of inadequate calorie and protein intakes as well as a low intake of iron and zinc. Similarly, Xie et al. [8] suggested that efforts to intensify sodium restriction might increase the risk of compromising energy intake further. This study found that patients with low corrected salt intake had lower nutrients such as BMI, nPCR, and serum albumin (shown in Table 2).

Nutritional status is associated with a risk of death and infection. A recently reported three-year prospective cohort study suggests that dietary intake, body composition, and bone metabolism are predictors of mortality in hemodialysis patients [18]. Toyoda et al. [19] reported that a decrease in serum albumin levels was associated with a higher risk of infectious disease and hospitalization. The present data showed that patients with low corrected salt intake were at increased risk of all-cause mortality and had a higher mortality due to infectious disease (shown in Figs 3 and 4). For the Q1 group, which had a low salt intake, the intake may have been too low to meet nutritional requirements. On the other hand, in the multivariate analysis model adjusted by nutritional factors, the association between the corrected salt intake and all-cause mortality remained (shown in Table 3). In this study, patients with lower salt intake had lower serum sodium concentrations (shown in Table 2), which may have influenced this study. Recent reports suggest that hyponatremia increases the risk of infection [20], and associations between hyponatremia and greater mortality from these diseases have also been identified. The mechanism involves mucosal edema that develops due to extracellular hypotonicity and osmosis into the intracellular space, which compromises the microbial barrier function of the mucosa in the respiratory, gastrointestinal, and urinary tracts [20]. In addition, hyponatremia has been reported to be associated with mortality [21], malnutrition [22], osteoporosis [23], cognitive dysfunction [24,25], and gait disturbance [26], all of which have a substantial influence on the prognosis of hemodialysis patients.

In a nested case-control study using sub-groups of the same age distribution, corrected salt intake of the non-survivor group was significantly lower than that of the survivor group. The above results also support the association between corrected salt intake and mortality. However, a retrospective observational study cannot completely rule out the effects of various factors, including nutrition. Intervention trials are needed to clarify optimal salt intake. We consider that salt intake is intrinsically linked to other forms of nutritional intake, and it is important to consider salt within the context of the patient’s overall pattern of dietary intake.

In dialysis patients, sodium intake is balanced with water intake because the kidneys are not functioning properly. Thus, salt intake is directly related to IDWG. Some previous studies have discussed the association between IDWG and cardiovascular events. Lee et al. examined the association between %IDWG and major adverse cardiac and cerebrovascular events, and reported %IDWG ≥ 4.0% was a significant predictor of primary outcome [5]. Similarly, a study of 34,107 hemodialysis patients across the United States suggested that greater fluid retention between 2 successive hemodialysis treatment sessions is associated with higher risk of 2-year all-cause and cardiovascular death [6]. On the other hand, in other studies, the association between salt intake and cardiovascular events was not as strong. In a large cohort study of the general population, a high sodium intake was found to be associated with an increased risk of cardiovascular events and death in hypertensive populations, while the association of low sodium intake with increased risk of cardiovascular events and death was also observed in those with or without hypertension [1]. In a study of Japanese dialysis patients, a low salt intake was associated with an increased risk of all-cause mortality and cardiovascular death [11]. The present study found no clear difference in the four-year cumulative incidence of cardiovascular events and mortality caused by cardiovascular disease among groups with correction for salt intake. However, in the multivariate analysis model adjusted for cardiovascular risk factors, %IDWG was positively associated with all-cause mortality. These findings suggest that it is not possible to simply correlate salt intake with IDWG and assess the effect of salt intake directly from the association between IDWG and mortality. Salt intake can be strongly associated with nutritional intake, which may be as important to dialysis patients as the increase in IDWG, which is a cardiovascular risk.

We used the corrected salt intake for IBW as an observation indicator in this study. IBW was calculated on the assumption that the ideal BMI is 22 kg/m2 for the Japanese population [13]. In hemodialysis patients, higher BMI is known to be associated with better survival [27]. On the other hand, recent studies have gone beyond BMI to further our understanding of the impact of body composition on survival and on other important outcomes. Johansen et al. reported higher fat mass and higher extracellular water was associated with higher odds of frailty in a cross-sectional analysis of 638 adult patients receiving maintenance hemodialysis [28]. Several studies have examined the association between the amount of visceral adipose tissue and outcomes, and reported that the presence of visceral obesity was associated with a higher coronary artery calcification score and higher risk of cardiovascular events [29,30]. Furthermore, it has been demonstrated that an excess of fat mass in obese patients can amplify the oxidative stress and inflammation caused by renal insufficiency [31]. These data suggest the negative metabolic consequences of excess fat are retained despite the association of higher BMI with better survival in dialysis patients. In this study, the mean BMI of the Q3 group with the highest survival rate was 22 kg/m^2^, which was similar to the ideal BMI of Japanese. This result suggests that the ideal BMI for dialysis patients remains controversial. Since salt intake is correlate with dietary intake, we consider that that salt intake should be corrected, at least in some way.

This study has some limitations. First, we were unable to assess smoking status. A previous study in 223,000 patients reported that risks for death and hospitalization are elevated among hemodialysis patients who smoke [32]. Second, we assumed that patients who underwent hemodialysis for more than 2 years developed anuria, but we were unable to accurately assess residual renal function at baseline. In addition, we had no data on dietary constituents, perspiration or physical activity. These factors may affect estimation of salt intake [15,16]. Therefore, it is possible that salt intake was underestimated. Third, we did not add pre-dialysis sodium concentration to the multivariate analysis model. Pre-dialysis sodium concentration has a strong relationship with mortality [21]. However, there was a strong positive correlation between pre-dialysis serum sodium concentration and corrected salt intake, and there was multicollinearity between them. Therefore, we determined that pre-dialysis sodium concentration was not suitable as a factor in the multivariate analysis model. Fourth, this study analyzed only a Japanese dialysis population. Thus, any effects on health outcomes of a diet peculiar to the Japanese, body type, and dialysis method were not verified. Trials of interventions are warranted to clarify the management of optimal salt intake.

In conclusion, the results of the present study showed that a low salt intake was associated with high 4-year all-cause mortality. Previous studies and our data suggest that excessive salt restriction may increase the risk of malnutrition and reduce long-term survival. When restricting salt in patients on hemodialysis it is important to consider this in the context of adequate nutritional intake.
